# MR-angiography allows defining severity grades of cerebral vasospasm in an experimental double blood injection subarachnoid hemorrhage model in rats

**DOI:** 10.1371/journal.pone.0171121

**Published:** 2017-02-09

**Authors:** Vesna Malinova, Marios N. Psychogios, Ioannis Tsogkas, Birte Koennecke, Kim Bleuel, Bogdan Iliev, Veit Rohde, Dorothee Mielke

**Affiliations:** 1 Department of Neurosurgery, Georg August University Göttingen, Germany; 2 Department of Neuroradiology, Georg August University Göttingen, Germany; 3 Department of Neurology, Georg August University Göttingen, Germany; Heinrich-Heine-Universitat Dusseldorf, GERMANY

## Abstract

**Objective:**

Magnetic resonance (MR) imaging has been used for the detection of cerebral vasospasm (VSP) related infarction in experimental subarachnoid hemorrhage (eSAH) in rats. Conventional angiography is generally used to visualize VSP, which is an invasive technique with a possible increase in morbidity and mortality. In this study we evaluated the validity of MR-angiography (MRA) in detecting VSP and its feasibility to define VSP severity grades after eSAH in rats.

**Methods:**

SAH was induced using the double-hemorrhage model in 12 rats. In two rats, saline solution was injected instead of blood (sham group). MR was performed on day 1, 2 and on day 5. T1-, T2-, T2*-weighted and time-of-flight MR sequences were applied, which were analyzed by two blinded neuroradiologists. Vessel narrowing of 25–50% was defined as mild, 50–75% as moderate and >75% as severe VSP.

**Results:**

We performed a total of 34 MRAs in 14 rats. In 14 rats, MRA was performed on day 2 and day 5. In six rats MRA was additionally performed on day1 before the blood injection. A good visualization of cerebral vessels was possible in all cases. No VSP was seen in the sham group neither on day 2 nor on day 5. We found vasospasm on day 2 in 7 of the 14 rats (50%) whereas all 7 rats had mild and one rat had additionally moderate and severe vasospasm in one vessel, respectively. On day 5 we found vasospasm in 8 of the 14 rats (60%) whereas 4 rats had severe vasospasm, 1 rat had moderate vasospasm and 3 rats demonstrated mild vasospasm. In 4 of the 14 rats (30%) an ischemic lesion was detected on day 5. Three of these rats had severe vasospasm and one rat had mild vasospasm. Severe vasospasm on day 5 was statistically significant correlated with the occurrence of ischemic lesions (Fisher’s Exact test, OR 19.5, p = 0.03).

**Conclusions:**

MRA is a noninvasive diagnostic tool, which allows a good visualization of the cerebral vasculature and provides reproducible results concerning the detection of VSP and the differentiation into three severity grades in rats. Future studies are needed to directly compare MRA with conventional angiography.

## Introduction

Cerebral vasospasm (VSP) is still one of the main reasons for delayed cerebral ischemia (DCI) after aneurysmal subarachnoid hemorrhage (aSAH). Different animal models of experimental subarachnoid hemorrhage (eSAH) in rats have been established during the last years [1–5]. The double hemorrhage model has been used for the investigation of delayed VSP [4]. In order to increase the incidence and severity of VSP, a modified double hemorrhage model of eSAH was recently introduced, in which unilateral occlusion of the common carotid artery is additionally performed [5]. Up to date, digital subtraction angiography (DSA) is the gold standard for the diagnosis of vasospasm and has been already applied in different experimental studies in rats [4,6]. But, DSA is an invasive method, possibly leading to an increase of morbidity and mortality rates. Therefore, noninvasive diagnostic methods are warranted, which likewise allow a reliable diagnosis of VSP and the differentiation of VSP severity grades as well.

Magnetic resonance (MR) imaging is a noninvasive technique, which is routinely used in experimental studies for the diagnosis of cerebral ischemia [4,5,7]. The aim of this study was to evaluate the utility of MR angiography (MRA) in the diagnosis of VSP in eSAH in rats using the modified double hemorrhage model. Furthermore, our aim was to define different VSP severity grades. To the best of our knowledge, this is the first study, which addresses the role of MRA in eSAH for VSP diagnosis and VSP grading.

## Materials and methods

### 1. Experimental set-up

All experiments were conducted in accordance with the “Guide for the Care and Use of Laboratory Animals of the NIH” and were ethically approved by the Government of Lower Saxony/Germany (AZ 13/1055).

The experiments were performed on 14 male Sprague Dawley rats (Charles River, Germany; body weight 250-320g). The animals were housed in a temperature- and humidity-controlled room on a 12-hour light/12-hour dark cycle in single cages under standard laboratory conditions, with food and water *ad libidum*.

Twelve rats were assigned to the eSAH group using the modified double hemorrhage model [5], and two rats to the sham group. Anesthesia was performed by intraperitoneal injection of an anesthetic cocktail (1ml) consisting of 0.3ml medetomidine (Cepetor^®^ 1mg/ml) and 0.7ml ketamine (Ketamin^®^ 10% Solution) with an applied dosage of 0.1ml per 100g body weight. Body temperature was maintained between 36.5 and 37.5°C by a heating blanket, controlled by a rectal temperature probe (Homeothermic Monitor, Harvard Apparatus, Hugo Sachs Elektronik, Germany).

The double hemorrhage model of eSAH included the injection of 0.25ml autologous arterial blood into the subarachnoid space on two consecutive days. Prior to the first blood injection, an occlusion of the left common carotid artery was performed in order to increase the predisposition for the development of VSP. The blood was drawn from the tail artery via a percutaneous puncture using a 27gauge cannula (B. Braun Melsungen AG, Melsungen, Germany). The blood into the subarachnoid space was administered through the cerebellomedullary cistern via a puncture of the posterior atlantooccipital membrane using a midline approach. For this purpose the rat was fixed on a stereotaxic frame. Prior to the blood injection, 0.1ml of cerebrospinal fluid were withdrawn. The blood was injected via a special thin and soft catheter (Portex polythene tube with luminal diameter of 0.28 mm) in order to avoid brainstem injury during the blood injection. After withdrawal of the catheter, the opening in the posterior atlantooccipital membrane was closed using a fibrillar hemostyptic material (Tabotamp, Ethicon, Somerville USA, Johnson&Johnson Medical, Norderstedt Germany) in order to avoid a leakage of the injected blood. After the blood injection, the rat was positioned in an approximately 20° head down position for 15 minutes, to allow a sufficient distribution of the injected blood into the subarachnoid space.

For the second blood injection on day 2, the blood was again drawn from the tail artery using a 27gauge cannula. The blood injection was performed via the same surgical approach as on day one. The wounds were closed with single button sutures. For postoperative pain control, all rats received buprenorphine (Temgesic^®^, RB Pharmaceuticals Limited, Berkshire, United Kingdom) (0.03–0.05μg/kg to 0.1μg/kg body weight s.c.) as well as 5ml saline solution s.c. twice a day and metamizole (1.33mg/ml drinking water p.o.) continuously. On day 5, a transcardial perfusion with phosphate buffered saline (PBS) and paraformaldehyde (4% solution) through a transthoracic approach was performed. In the sham group 0.25ml saline solution instead of autologous blood was injected on two consecutive days. There were no other differences between the eSAH and sham group. The rats were weighed daily.

#### Health status of the animals and mortality rate

The mortality rate was 52% (16/30), which is in line with the mortality rates described by Güresir et al. and Vatter et al. who developed this eSAH model for research on delayed vasospasm. 15% of the rats died on day 1 after the first blood injection and 35% on day 2 after the second blood injection. There was no mortality after day 2. The animals that survived until day 5 were included in this study.

On day 1 and 2 we observed a weight loss of 10–15% of the initial weight in all rats. They gained again weight on day 3–5. None of the rats had motor deficits. The rats with more severe SAH showed less movement, especially on day 1 and 2 and recovered continuously until day 5. We did not find secondary clinical deterioration on day 3, 4 or 5.

### 2. Imaging protocol

All MR images were acquired on a 3.0-T scanner (Magnetom TIM Trio, Siemens, Erlangen, Germany). Rats were placed in a circular 8-channel wrist coil (Siemens, Erlangen, Germany) on day 1 prior to the first blood injection, on day 2 after the second blood injection and on day 5 prior to transcardial perfusion. The coil was placed inside the MR scanner with the longitudinal axis of the rat roughly parallel to B0. Sequence parameters are summarized in Table 1. The duration of a single examination was 45 minutes.

**Table 1:** Magnetic resonance imaging parameters.

	T1	T2	3D-TOF	T2*
TR (ms)	2250	2900	15	31
TE (ms)	4,06	233	6,15	25,8
Flip angle (degrees)	9	120	23	8
Matrix size (number of data points)	320 x 130	320 x 130	448 x 294	384 x 108
Slice thickness (mm)	0,33	0,4	0,33	0,33
Bandwidth (pixel)	355	200	100	310

TR = repetition time;

TE = echo time

### 3. Image analysis

**Fig 1:**
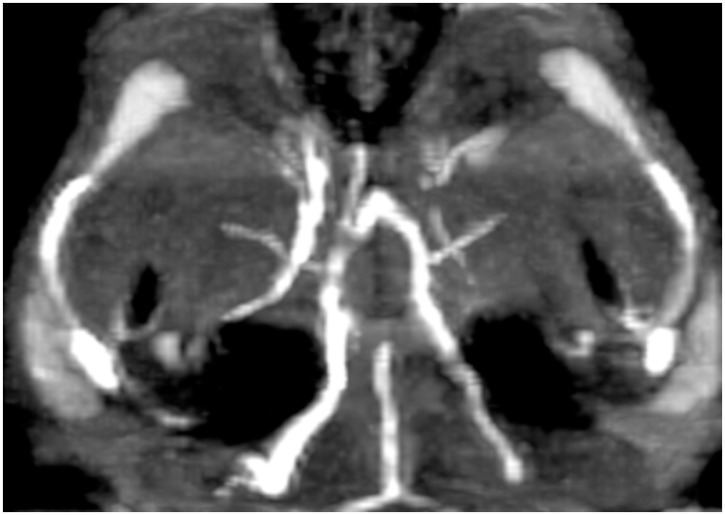
Mild vasospasm of A1 segment of the right anterior cerebral artery.

Two experienced neuroradiologists analyzed the MRI datasets on a syngo X-workplace (Siemens, Erlangen, Germany) to allow interactive reconstruction and interpretation. Both raters were blinded for time point and injection information (experimental group versus sham). All major basal cerebral arteries (anterior cerebral artery, middle cerebral artery, internal carotid artery and the basilar artery) were evaluated for the presence of caliber variations indicating VSP. Ischemia was defined as a new hyperintense lesion on T2 weighted MR images. The vessel diameter measurements were performed after reconstruction of the dataset of the TOF-sequence in 3mm MIP. We measured all vessels and, in case of a caliber change, we measured the most severe stenosis of the affected vessel. The median diameter of the internal carotid artery on the MRA on day 1 was 0.7mm, of the anterior cerebral artery, middle cerebral artery and basilar artery 0.6mm. Because we did not perform MRI on day 1 in all rats, we compared the measurements on day 2 and day 5 with the median diameter of the vessels on day 1. For grading the VSP severity, the vessel diameter of these arteries was measured (Fig 1). The baseline data served as reference values for the diagnosis of VSP. A vessel narrowing of 25–50% was classified as mild VSP, of 50–75% as moderate VSP and of >75% as severe VSP.

### 4. Statistical analysis

For the evaluation of the incidence of VSP descriptive statistic methods were used (GraphPad Software, La Jolla USA). The absolute and relative frequency distribution for the occurrence of VSP and of the three severity grades was calculated on day 2 and day 5, respectively.

## Results

**Fig 2:**
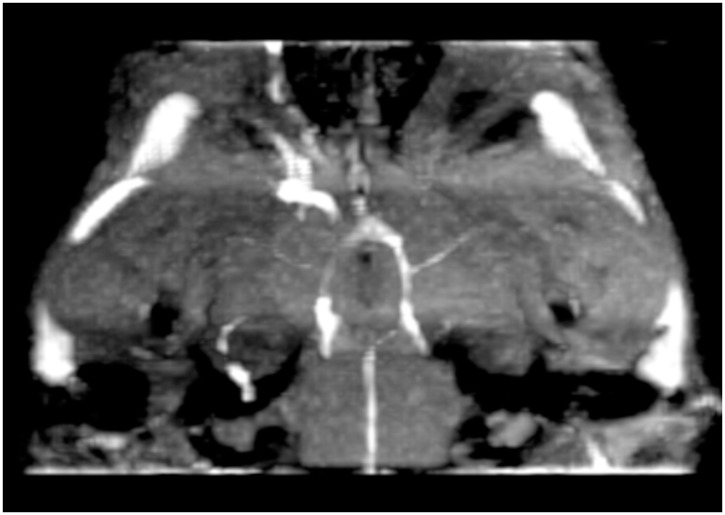
Severe vasospasm of both M1 segments of the middle cerebral artery.

**Fig 3:**
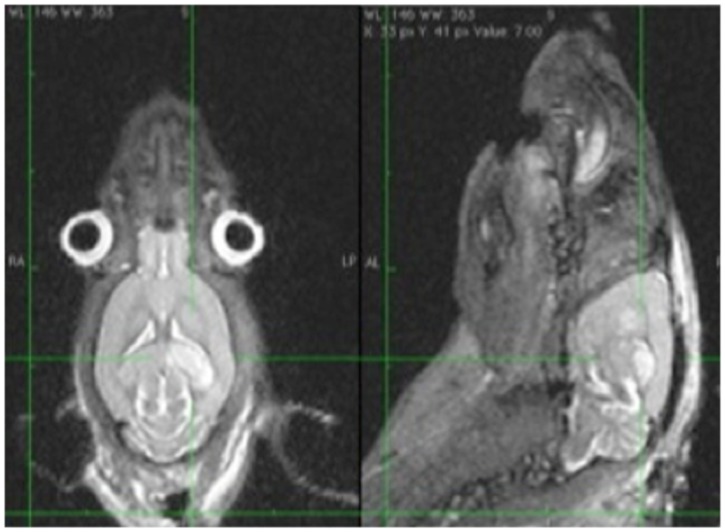
Ischemic lesion of the left thalamus.

A total of 34 MRA scans were performed in 14 rats. Twelve rats were in the eSAH group and two rats in the sham group. All 14 rats underwent MRA on day 2 and on day 5. In six rats MRA was additionally performed on day1 before the blood injection. All 34 MRA scans allowed a good visualization of the circle of Willis. We found vasospasm on day 2 in 7 of the 14 rats (50%) whereas all 7 rats had mild and one rat had additionally moderate and severe vasospasm in one vessel territory, respectively. In four of these 7 rats only one vessel was affected by vasospasm, in one rat three vessels and in two rats vasospasm was seen in four vessels simultaneously. On day 5 we found vasospasm in 8 of the 14 rats (60%), whereas 4 rats had severe vasospasm, 1 rat had moderate vasospasm and 3 rats had mild vasospasm. Out of the four rats with severe vasospasm, three had severe vasospasm in two vessels simultaneously and one rat had severe vasospasm in one vessel and additionally moderate vasospasm in one vessel and mild vasospasm in one vessel, respectively. We found vasospasm neither on day 2 nor on day 5 in the two sham-group rats. The median diameter of the internal carotid artery on the MRA on day 1 was 0.7mm. The median diameter of the anterior cerebral artery, middle cerebral artery and basilar artery was 0.6mm. In 4 of the 14 rats an ischemic lesion was seen on the MRI scan on day 5. All four rats had vasospasm on day 5, whereas three of these rats had severe vasospasm and one rat had mild vasospasm. The rats with vasospasm on the MRA scan on day 5 had a 2.5 fold higher risk to develop ischemic lesions compared to the rats without vasospasm (Fisher’s Exact test, OR 2.5 p = 0.08). Severe vasospasm on day 5 was statistically significantly correlated to the occurrence of ischemic lesions (Fisher’s Exact test, OR 19.5, p = 0.03). The ischemic lesions were located mostly in the thalamus, the basal ganglia or in the hippocampus. Two rats had more than one ischemic lesion at the same time. The results are summarized in Table 2. The measurements of the vessel’s diameter are shown in Fig 1 (as an example of the MCA). Examples of mild and severe VSP and of an ischemic lesion are shown in Figs 1, 2 and 3, respectively. Tables 3 and 4 show the quantitative analysis of the vessel diameter of each vessel on day 1, day 2 and day 5, respectively.

**Table 2:** Vasospasm (VSP) incidence and differentiation of mild/moderate/severe VSP and ischemic lesions.

Rat group	VSP day 2	Location and grade	VSP day 5	Location and grade	Ischemic lesion	Location
**SAH**	no	/	yes	left+right MCA—severe	yes	left+right thalamus
**SAH**	yes	right MCA—mild	yes	right ACA—severe right MCA—moderate left ICA—mild	no	/
**SAH**	yes	right ACA—mild left+right MCA—mild BA—mild	yes	right ACA—mild left MCA—mild	yes	right thalamus
**SAH**	no	/	no	/	no	/
**SAH**	no	/	no	/	no	/
**SAH**	yes	left MCA—mild	yes	left+right MCA—severe	yes	left basal ganglia
**SAH**	no	/	no	/	no	/
**SAH**	yes	left+right ACA—mild left ICA—mild	yes	left+right ACA—mild left MCA—mild	no	/
**SAH**	yes	left ICA—mild	yes	left+right MCA—mild	no	/
**SAH**	yes	left ACA—mild right MCA—mild left MCA—moderate left ICA—severe	yes	left+right MCA—mild	no	/
**SAH**	no	/	no	/	no	/
**SAH**	yes	right MCA—mild	yes	left MCA—severe left MCA—severe	yes	left hippocampus left thalamus left basal ganglia
**Sham**	no	/	no	/	no	/
**Sham**	no	/	no	/	no	/

ACA = anterior cerebral artery, MCA = middle cerebral artery, ICA = internal carotid artery, BA = basilar artery

**Table 3:** Quantitative analysis of the vessel diameter of the basal cerebral arteries on day 1 and day 2.

	BA day 1	ACI day 1	MCA day1	ACA day 1	BA day 2	ACI day 2	MCA day 2	ACA day 2
Mean	0.616	0.683	0.608	0.6	0.59	0.65	0.57	0.58
Median	0.6	0.7	0.6	0.6	0.6	0.7	0.6	0.6
IQR	0.6–0.6	0.7–0.7	0.6–0.6	0.6–0.6	0.6–0.6	0.6–0.7	0.5–0.6	0.6–0.6
SD	0.040	0.038	0.028	0	0.049	0.12	0.06	0.03
SE	0.01	0.011	0.008	0	0.013	0.024	0.013	0.007
Range	0.6–0.7	0.6–0.7	0.6–0.7	0.6–0.6	0.5–0.7	0.1–0.7	0.4–0.7	0.5–0.6
CoK	6 (p:0,0076)	2.64 (p:0.072)	12 (p:0001)	0	2.57 (p:0.07)	16.37 (p<0.0001)	0.78 (p:0.30)	2.32 (p:0.044)
RSD	6.62%	5.7%	4.75%	0	8.33%	19.09%	11.64%	6.29%

SD: standard deviation, SE: standard error of the mean, CoK: coefficient of Kurtosis, RSD: Relative standard deviation, ACI: internal carotid artery, ACA: anterior cerebral artery, BA: basilar artery, MCA: middle cerebral artery; BA: basilar artery (das muss noch nach Alphabet sortiert werden)

**Table 4:** Quantitative analysis of the vessel diameter of the basal cerebral arteries on day 5.

	BA day 5	ACI day 5	MCA day 5	ACA day5
Mean	0.57	0.628	0.485	0.5286
Median	0.6	0.6	0.6	0.6
IQR	0.5–0.6	0.6–0.7	0.5–0.6	0.5–0.6
SD	0.04	0.053	0.1693	0.1357
SE	0.012	0.010	0.032	0.0256
Range	0.5–0.6	0.5–0.7	0.1–0.6	0.1–0.6
CoK	-1.03 (p:0.307)	-0.420 (p:0.707)	0.4616 (p:0.452)	5.9825 (p:0.0011)
RSD	8.20%	8.50%	34.86%	25.67%

SD: standard deviation, SE: standard error of the mean, CoK: coefficient of Kurtosis, RSD: Relative standard deviation, ACI: internal carotid artery, ACA: anterior cerebral artery, BA: basilar artery, MCA: middle cerebral artery

## Discussion

In this study, we investigated the utility of MRA for the detection of experimental VSP as well as the possibility to distinguish between different severity grades of VSP in a double hemorrhage eSAH-model in rats. As rats compared to humans have a good collateral blood supply of the brain and a higher metabolism rate allowing a faster clearance of the blood from the subarachnoid space, it is more difficult to induce vasospasm by a single blood injection. To increase the rate of delayed vasospasm, blood was injected on two consecutive days and additionally the left common carotid artery has been occluded, thereby reducing the tolerance to ischemia in the rats. As a consequence, the modified double blood injection model is associated with a high mortality rate, but allows better than other models the investigation of delayed vasospasm.

The major findings of our study are that 1- MRA is a reliable noninvasive technique that allows the diagnosis of VSP and that 2- a differentiation between three severity grades of VSP is actually possible.

The visualization of the cerebral vasculature is of a great importance for the investigation of VSP. DSA is considered to be the gold standard for the evaluation of VSP after aSAH in humans as well as in eSAH. But, DSA is an invasive and elaborate technique with a risk for permanent or transient neurological deficits of up to 3.7% in humans [8]. Thus, it can be assumed that DSA might contribute to the high mortality rates in eSAH. In humans, MRA is an alternative noninvasive method for the detection of VSP after aSAH with a high sensitivity and specificity and a good correlation compared to DSA [9]. The visualization of cerebral vessels in eSAH models in rodents is challenging because of the small vessel diameter. Previous studies mentioned the possibility to measure the diameter of cerebral vessels in different eSAH-models in rats with selective conventional angiography as well as by histological means [4,5]. Most previous studies focused on the measurement of the diameter of the basilar artery. Reports on the diameter of other cerebral vessels are rare. Vatter et al. examined the time course of the development of angiographic VSP in a double hemorrhage eSAH-model in rats using selective DSA of the posterior circulation via the vertebral artery [4]. For the diagnosis of angiographic VSP the diameter of the basilar artery (BA) was measured. They found a reduction in diameter of the BA using DSA on day 2 and 3, but the greatest reduction was seen on day 5 [4]. Vessel diameter and blood flow velocity were calculated on the basis of MRA in an endovascular perforation eSAH-model in rats by Van den Bergh et al. Examinations were done 2 hours, 2 days and 9 days after the induction of eSAH [10]. They provided data of the vessel diameter of all basal cerebral arteries. They found no difference in vessel diameter two hours after SAH compared to the sham group. On day two after SAH the vessel diameter was smaller and on day 9 they found normal vessel diameter again [10]. In this study, we detected a mild reduction in vessel diameter also on day 2 and a severe vessel narrowing of >75% on day 5 after SAH. We found a significant correlation of severe vasospasm with the occurrence of ischemic lesions on day 5. In three out of four rats with ischemic lesions severe vasospasm was seen on the MRA on day 5. As outlined above, the occlusion of the left common carotid artery in the modified double blood hemorrhage SAH model increases the incidence of vasospasm-associated ischemic lesions in areas being more sensitive to hypoxia [5]. We found ischemic lesions only on day 5 and no ischemic lesions on day 2. These findings support the hypothesis that ischemic lesions indeed are the consequence of the visualized severe vasospasm on day 5.

Up to date, there are no conclusive data about different severity grades of VSP after eSAH. We have used the modified double hemorrhage eSAH-model, because of the high incidence of delayed VSP [5]. Gules et al have performed a comparison between the three most common eSAH-models in rats including the endovascular perforation model, the single-hemorrhage model and the double hemorrhage model [11]. They measured the perimeter of the BA and the posterior communicating artery and the histological thickness of the vessel wall. The authors found a significant increase in vessel wall thickness of the BA and a smaller luminal perimeter in the double hemorrhage model compared to the other eSAH-models indicating that the modified double hemorrhage eSAH model might be superior for delayed VSP research [11].

All of the mentioned eSAH-models showed a reproducible induction of VSP, but neither one of them was able to establish criteria for the differentiation between mild, moderate and severe VSP. A possible explanation could be the lower incidence of severe VSP in other SAH models compared to the modified double hemorrhage model. The differentiation of different severity grades of VSP is important concerning the efficacy of therapeutic research approaches for VSP. By using the double-blood-injection model [5] we were able to demonstrate that it is possible to distinguish between mild, moderate and severe VSP using MRA. The high mortality rate of the model is without doubt a disadvantage of the modified double blood injection model. However, this model yields a high incidence of severe delayed vasospasm and, at the end, might reduce the number of animals needed to operate to perform a meaningful statistical analysis. Hence, we decided to use this eSAH model in this study.

We believe that MRA might be an appropriate noninvasive alternative to DSA and the basis for future studies on VSP after experimental SAH in rodents. Future studies with larger number of examinations are needed to show the actual diagnostic value of MRA. Besides, studies comparing the results of MRA with DSA as well as histological analysis should be initiated. MRA for the diagnosis of cerebral vasospasm after SAH in humans has the limitation of a longer scanning time if compared with CT angiography. Additionally, in humans with SAH, invasive monitoring with intracerebral probes is often used with a subsequent contraindication for MRI and MRA. However, at least in good grade patients, MRA could be a viable alternative to CT angiography, which has the disadvantage of a considerable radiation exposure especially if used repeatedly during the vasospasm period.

### Limitations and strengths of the study

In this study, we focused on the technical feasibility of MRI/MRA in order to demonstrate and evaluate VSP after eSAH. We used a sham group, but did not include a control group, comparing MRA data with DSA. Another limitation is the small number of examinations included in the study, especially the small number of sham animals not allowing statistical analysis in comparison to the SAH animals. Also a correlation of the occurrence of vasospasm and cerebral ischemia with parameters such as blood pressure and intracerebral pressure was not possible, because these parameters were not measured. These aspects have to be addressed in future experiments. Vasospasm is one of multiple possible factors leading to delayed cerebral ischemia after subarachnoid hemorrhage. Previously published studies reported that the treatment of vasospasm alone does not necessarily translate into better outcome of the patients [12,13]. The analysis performed in this study does not allow the differentiation if the ischemic lesions were indeed caused by the visualized vasospasm and/or by other possible causes of delayed cerebral ischemia after subarachnoid hemorrhage such as microthrombosis, cortical spreading depression or neuroinflammation [14].

The strength of the study is in the definition of reproducible criteria for the diagnosis of VSP within the circle of Willis and for the differentiation between VSP severity degrees.

## Conclusion

MRA is a useful noninvasive diagnostic tool that enables the reliable diagnosis of cerebral VSP in an eSAH-model in rats. Furthermore, MRA allows differentiating between mild, moderate and severe VSP. Future studies are needed comparing MRA with DSA.
